# Epistatic Interactions Between Mutations of Deoxyribonuclease 1-Like 3 and the Inhibitory Fc Gamma Receptor IIB Result in Very Early and Massive Autoantibodies Against Double-Stranded DNA

**DOI:** 10.3389/fimmu.2018.01551

**Published:** 2018-07-05

**Authors:** Thomas Weisenburger, Bettina von Neubeck, Andrea Schneider, Nadja Ebert, Daniel Schreyer, Andreas Acs, Thomas H. Winkler

**Affiliations:** ^1^Department of Biology, Nikolaus-Fiebiger-Center for Molecular Medicine, Friedrich-Alexander-University Erlangen-Nuremberg, Erlangen, Germany; ^2^Medical Immunology Campus Erlangen, Friedrich-Alexander-University Erlangen-Nuremberg, Erlangen, Germany

**Keywords:** Dnase1l3, anti-DNA autoantibodies, systemic lupus erythematosus, Fcgr2b, germinal center

## Abstract

Autoantibodies against double-stranded DNA (anti-dsDNA) are a hallmark of systemic lupus erythematosus (SLE). It is well documented that anti-dsDNA reactive B lymphocytes are normally controlled by immune self-tolerance mechanisms operating at several levels. The evolution of high levels of IgG anti-dsDNA in SLE is dependent on somatic hypermutation and clonal selection, presumably in germinal centers from non-autoreactive B cells. Twin studies as well as genetic studies in mice indicate a very strong genetic contribution for the development of anti-dsDNA as well as SLE. Only few single gene defects with a monogenic Mendelian inheritance have been described so far that are directly responsible for the development of anti-dsDNA and SLE. Recently, among other mutations, rare null-alleles for the deoxyribonuclease 1 like 3 (*DNASE1L3*) and the Fc gamma receptor IIB (*FCGR2B*) have been described in SLE patients and genetic mouse models. Here, we demonstrate that double Dnase1l3- and FcgR2b-deficient mice in the C57BL/6 background exhibit a very early and massive IgG anti-dsDNA production. Already at 10 weeks of age, autoantibody production in double-deficient mice exceeds autoantibody levels of diseased 9-month-old NZB/W mice, a long established multigenic SLE mouse model. In single gene-deficient mice, autoantibody levels were moderately elevated at early age of the mice. Premature autoantibody production was accompanied by a spontaneous hyperactivation of germinal centers, early expansions of T follicular helper cells, and elevated plasmablasts in the spleen. Anti-dsDNA hybridomas generated from double-deficient mice show significantly elevated numbers of arginines in the CDR3 regions of the heavy-chain as well as clonal expansions and diversification of B cell clones with moderate numbers of somatic mutations. Our findings show a strong epistatic interaction of two SLE-alleles which prevent early and high-level anti-dsDNA autoantibody production. Both genes apparently synergize to keep in check excessive germinal center reactions evolving into IgG anti-dsDNA antibody producing B cells.

## Introduction

The formation of antibodies against DNA is considered to be the serologic hallmark of systemic lupus erythematosus (SLE). Autoantibodies against double-stranded DNA (anti-dsDNA) antibodies are the most studied and the most enigmatic autoantibodies in SLE. Their presence correlates with nephritis both in human SLE patients and in mice with a spontaneous lupus-like disease (1–3).

Systemic lupus erythematosus has a strong genetic component as demonstrated by high concordance rates for disease manifestation as well as autoantibody development in monozygotic twins (4, 5). In rare cases, a monogenic cause for the development of SLE-like disease and autoantibody production illustrates the pathways that predispose for SLE. The strongest associations were found to loss of function of genes that are involved in clearance of apoptotic cells, chromatin, and nucleic acids (6). Homozygous mutations in the *C1q* genes are responsible for SLE or SLE-like disease (7) and *C1q*-deficient mice accumulate apoptotic cells in several tissues and develop anti-DNA autoantibodies and SLE in certain genetic backgrounds but not in the C57BL/6 background (8, 9). Homozygous mutations in the *TREX1* gene encoding a DNA exonuclease present in the cytoplasm are associated with the Aicardi–Goutieres syndrome-1 with increased systemic type I interferon levels and antinuclear autoantibodies (10). Likewise, *Trex1*-deficient mice develop autoantibodies and SLE-like disease.

Other apparently monogenic causes of SLE-like disease and anti-DNA autoantibodies were described in mutant mice. The deficiency of the IgG inhibitory Fc γ receptor IIB (FcγRIIB) is associated with the development of SLE in mice with a strong influence of background genes, however (11, 12). In the absence of FcγRIIB, autoreactive B cells are found in the germinal centers and somatic hypermutation contributed to anti-DNA reactivity (13). In mice, a promoter variant in the *Fcgr2b* gene results in enhanced germinal center responses and autoantibody production (14). A defunctioning single nucleotide polymorphism (SNP) in the human *FCGR2B* gene is associated with susceptibility to SLE (15) and the upregulation of FcγRIIB in memory B cells is decreased in SLE patients (16). In addition, FcγRIIB plays an important regulatory function on dendritic cells (DCs), macrophages, and neutrophils [reviewed in Ref. (17)].

Another single gene defect leading to anti-DNA production and SLE-like disease is demonstrated by the observation that milk fat globule-EGF factor 8 has a critical role in removing apoptotic B cells in the germinal centers and that its absence can lead to autoimmune diseases (18).

More recently, deficiency in the *Deoxyribonuclease 1 like 3* gene (*DNASE1L3*) was associated with SLE-like disease in Arabian, Turkish, and Italian families (19–21). SLE-like symptoms were associated with a hypocomplementemic urticarial vasculitis syndrome in several clinical cases. In *Dnase1l3-*deficient mice, anti-dsDNA autoantibodies develop, followed by a late onset of SLE-like disease (22). Dnase1l3 is part of four homologous mammalian extracellular deoxyribonucleases of the Dnase1 family, Dnase1, Dnase1l1, Dnase1l2, and Dnase1l3 (23).

Here, we describe a new conditional mouse model for Dnase1l3 deficiency and confirm the early appearance of IgG anti-dsDNA autoantibodies at moderate levels in the serum of these mice. The development of anti-dsDNA antibodies does not require the presence of toll-like receptor 9 (TLR9) but a strong epistatic interaction with a mutation in the Fcgr2b gene leads to strikingly early and high anti-dsDNA serum levels accompanied by spontaneous hyperactivation of germinal centers.

## Materials and Methods

### Mice

Mice were maintained in SPF conditions at the animal facility of the Friedrich-Alexander-University, Erlangen. C57BL/6 mice were obtained from Charles River, Sulzfeld, Germany. TLR9-deficient mice (24) were a kind gift of H. Wagner, Munich. Fcgr2b-deficient mice (25) backcrossed for 12 generations to C57BL/6 were obtained from Taconic and the *yaa* mutation was introduced by breeding in the *yaa* mutation from male B6.SB-Yaa/J mice obtained from the Jackson laboratory. For the generation of Dnase1l3-deficient mice, a targeting vector was obtained from the KOMP consortium (project CSD48807, clone HTGRS01006_A_B09) and partially re-sequenced. Standard techniques were used to transfect the V6.5 embryonic stem cell line (kindly provided by R. Jaenisch, Boston) derived from a C57BL/6x129Sv F1 cross (26). Homologous recombination was screened with left arm and right arm primers by PCR using LongAmp^®^ Taq (New England Biolabs) according to the instructions of the manufacturer. Primers are listed in the Primer List in Data Sheet S2 in Supplementary Material. Correct integration was further verified by sequencing of the PCR products from both sides of the genomic integration. ES cells were injected into C57BL/6 blastocysts and chimeric mice were crossed with C57BL/6 mice. After germline transmission, one line was kept on C57BL/6x129sv mixed background. A pure backcross to C57BL/6 background was obtained by marker-assisted speed-congenics using 74 genomic SNP markers (LGC Genomics, KASP genotyping) covering all mouse chromosomes. After five generations, heterozygous offspring mice containing all marker loci from C57BL/6 were intercrossed to obtain C57BL/6 “knockout-first” Dnase1l3-deficient mice. After crossing with flp-deleter mice (27), Dnase1l3-deficient mice with a deleted exon 2 were created. For the establishment of Dnase1l3/Fcgr2b double-deficient mice, male Fcgr2b^−/−^ *yaa* mice were intercrossed with C57BL/6 “knockout-first” Dnase1l3-deficient mice. In the F2 generation, homozygous Dnase1l3/Fcgr2b double-deficient male and female mice were selected and used for further breeding. Animal care and experiments were approved by the animal ethics committee of Regierung von Mittelfranken (Animal Ethics Number: 54-2532.1-21/09 and TS-05/5).

### Flow Cytometric Analysis

For cell sorting of macrophage and DC cell populations, spleen, liver, lung, brain, and lamina propria tissue from 8- to 14-week-old C57BL/6 mice was mechanically dissociated with a gentle MACS™ and the respective tissue dissociation kit (Miltenyi Biotec). Bone marrow cells were flushed from femurs of C57BL/6 mice. For analysis of germinal center cells and myeloid cells, spleens were harvested, crushed through a 100 µM nylon mesh filter and resuspended in RPMI 1640 medium (PAN-Biotech) supplemented with 10% FCS. After erythrocyte lysis (5 min at room temperature in 0.15 M NH_4_Cl, 0.02 M HEPES, 0.1 mM EDTA), cells were washed two times and resuspended in FACS buffer (PBS, 2% FCS, 1 mM EDTA) for staining with directly conjugated antibodies. For intracellular IFN-γ staining, cells were treated with Cell Stimulation Cocktail + Protein Transport Inhibitors (eBioscience) for 2 h at 37°C. Cells were stained extracellularly for T_FH_ markers and were fixed and permeabilized with the FIX & PERM Kit (Nordic-MUbio) according to the manufacturer’s protocol. The following antibody conjugates were obtained from BioLegend: CD45-BV421, CD19-BV421, CD38-PE, GL7-APC, CD95-PECy7, CD11b-BV510, CD11b-PECy7, CD11c-APCCy7, LY6C-PerCPCy5.5, LY6G-FITC, CXCR5-BV421, and PD1-PECy7. The following antibody conjugates were obtained from eBioscience: MHCII-PE, CD4-FITC, CD44-APC, CD8-FITC, B220-PerCPCy5.5, IgD-PE, IgM-FITC, CD21-FITC, CD23-PE, and F4/80-AF647. BV510-conjugated anti-PSGL-1 and BV421-conjugated anti-F4/80 were obtained from BD Biosciences. APC-conjugated anti-Siglec-H was obtained from Miltenyi Biotec. Flow cytometry analysis was performed on a Cytoflex instrument (Beckman Coulter) and FlowJo v10.4 analysis software (FlowJo, LLC).

### ELISA for Anti-dsDNA Autoantibodies

Levels of anti-dsDNA were measured by ELISA. 20 µg/ml % poly-l-lysine (Sigma-Aldrich) was used to precoat MaxiSorp plates (Nunc). After 2 h at RT, plates were coated with dsDNA from calf thymus (20 µg/ml; Sigma-Aldrich) in TE (pH 7.5) buffer overnight at 4°C. Plates were washed with PBS/0.05% Tween 20 and sera was added in 1/2 serial dilutions starting at 1/100. A serum pool obtained from 9-month-old diseased NZB/W mice served as internal standard. The starting dilution of 1/200 of the NZB/W serum was arbitrarily assigned to 100 relative units. Goat-anti-mouse IgG or goat-anti-mouse IgM, Fc-specific, coupled to horseradish peroxidase was used for detection (Jackson ImmunoResearch).

### Gene Expression by Quantitative RT-PCR (qRT-PCR)

For qRT-PCR, RNA from FACS sorted cells was isolated by the RNeasy kit (QIAGEN) according to the manufacturer’s instructions. cDNA was synthesized by SCRIPTUM reverse transcriptase (Bio&Sell), and qRT-PCR was performed on a quantitative PCR system (Biorad CFX96) with Absolute qPCR SYBR Green Mix 2× (Thermo Scientific) using intron-spanning primers for Dnase1l3 and HPRT (see Primer List in Data Sheet S2 in Supplementary Material). In a second set of experiments, TaqMan^®^ Assays for Dnase1l3 and GAPDH were performed on an Applied Biosystems 7500 machine. Calculation of relative expression levels were performed according to the relative ΔCt-method (28).

### Hybridoma Production and Sequencing of V Region Genes

Hybridomas were generated from three female double-deficient mice that previously had a rise of IgG anti-dsDNA autoantibodies in the serum. As a fusion partner, SP2/0 cells obtained originally from F. Melchers, Basel were used. Standard techniques were applied and hybridoma supernatants were tested on day 10 after fusion for IgG anti-dsDNA antibodies by ELISA. Hybridoma cells from positive wells were expanded and subcloned by single cell sorting at least once.

Total RNA was isolated from the hybridomas with RNeasy (Qiagen) and variable genes of the hybridomas were cloned with RT-PCR using universal V_H_- and V_L_-primers (see Primer List in Data Sheet S2 in Supplementary Material) and the StrataClone PCR Cloning Kit. Inserts were Sanger sequenced with M13 primers annealing to the plasmid vector (LGC Genomics). The analysis for VH and VL gene usage, CDR-assignment and for potential somatic mutations was performed by V-QUEST (29) at the IMGT website.^1^ Sequence alignment was created with the Geneious Software (Biomatters Ltd.).

## Results

### Expression of *Dnase1l3* in Hematopoietic Cells

It was described that *Dnase1l3* is primarily expressed in macrophages in liver and spleen (30). To obtain a more detailed view of the expression pattern of *Dnase1l3* in hematopoietic cells in different tissues, we isolated lymphocyte, monocyte, macrophage, and DC populations from blood, bone marrow, spleen, lymph node, peritoneal cavity, liver, lung, brain, and lamina propria of the colon by FACS sorting and analyzed expression of *Dnase1l3* by quantitative real-time PCR. The results are summarized in Table 1. Among lymphocytes, a low-level expression of *Dnase1l3* was found in B1a B cells and marginal zone B cells, whereas conventional B cells as well as T cells did show no detectable expression. We did not detect any expression of *Dnase1l3* in different blood or tissue monocytes, including inflammatory monocytes from mice infected with the murine cytomegalovirus. Also, granulocytes did not exhibit detectable expression. As described before (22), conventional DCs in the spleen but also in liver and lamina propria expressed the highest levels of *Dnase1l3*. Among tissue resident macrophages, we detected a more complex expression pattern. Red pulp macrophages from spleen, Kupffer cells from liver, as well as macrophages from the lamina propria expressed very high levels, whereas macrophages isolated from the bone marrow, the peritoneum, the brain (microglia), and lung (alveolar macrophages) showed no detectable expression. Lower levels were detected in subcapsular sinus macrophages as well as plasmacytoid DCs. In summary, we detected Dnase1l3 expression mainly in antigen-presenting cell populations like conventional DCs and B1a and marginal zone B cells as well as in some tissue resident macrophages.

**Table 1:** Expression levels of Dnase1l3 in mouse hematopoietic cells.

Cell population; organ	Phenotype used for FACS sorting	Relative expression levels^a^
Pre/pro B cells, bone marrow	B220^+^ IgM^−^	–
Immature B cells, bone marrow	B220^+^ IgM^+^ IgD^−^	–
Follicular B cells, spleen	B220^+^ CD21^lo^ CD23^hi^	–
Marginal zone B cells, spleen	B220^+^ CD21^hi^ CD23^lo^	+
B1a B cells, peritoneum	B220^+^ CD11b^+^ CD5^+^	+
T cells, spleen	B220^−^ CD3^+^	–
Monocytes, non-classical, blood	CD45^+^ CD11b^+^ Ly6C^+^ MHCII^lo^	–
Monocytes, classical, blood	CD45^+^ CD11b^+^ Ly6C^−^ MHCII^lo^	–
Monocytes, peritoneum	CD45^+^ CD11b^hi^ F4/80^lo^ MHCII^+^	–
Monocytes, spleen	CD45^+^ CD11b^+^ Ly6C^+^ MHCII^lo^	–
Monocytes, lung	CD45^+^ CD11b^+^ LY6C^+^ MHCII^lo^	–
Monocytes, bone marrow	CD45^+^ CD11b^+^ Ly6C^+^ MHCII^lo^	–
Monocytes, lung, mCMV infection	CD45^+^ CD11b^+^ LY6C^+^ MHCII^+^	–
Neutr. granulocytes, bone marrow	CD45^+^ Ly-6G^hi^	–
Plasmacytoid dendritic cells (DCs)	CD45^+^ CD11c^lo^ SiglecH^+^	+
Myeloid DCs, spleen	CD11c^++^ MHC^++^ CD11b^+^ CD8^−^	+++
Lymphoid DCs, spleen	CD11c^++^ MHC^++^ CD11b^−^ CD8^+^	+++
DCs, liver	CD11b^+^ CD11c^+^ Ly6C^−^ MHCII^++^	++
DCs, lamina propria colon	CD11b^+^ CD11c^+^ Ly6C^−^ MHCII^++^	+++
Microglia, brain	CD45^lo^ F4/80^+^ CD11b^+^	–
Macrophages, bone marrow	CD45^+^ F4/80^+^ CD11b^low^ CD11c^−^	–
Macrophages, peritoneum	CD45^+^ F4/80^++^ CD11b^+^ CD11c^lo^	–
Alveolar macrophages, lung	CD45^+^ F4/80^+^ CD11b^low^ CD11c^++^	–
Macrophages, spleen	CD45^+^ F4/80^+^ CD11b^low^ CD11c^−^	+++
Kupffer cells, liver	CD45^+^ F4/80^+^ CD11b^low^ CD11c^−^	+++
Macrophages, lamina propria colon	CD45^+^ F4/80^+^ CD11b^low^ CD11c^−^	++
Subcapsular sinus macrophages, LN	CD11b^+^ CD169^+^ CD11c^−^ F4/80^−^	+

*Relative expression levels as determined by quantitative real-time PCR from sorted cells. Summary of two to five individual experiments, each*.

*^a^Relative expression levels as compared to expression levels in spleen CD11c^hi^ DCs*.

*–, not detectable; +, 1–5% of DCs; ++, 5–20% of DCs; +++, 20–100% of DCs*.

### Generation of Conditional Dnase1l3-Deficient Mice

To study the physiological function of Dnase1l3, we generated Dnase1l3-deficient mice using the “knockout-first” strategy as described by Skarnes et al. (31). The knockout-first allele is initially a non-expressive form, but can be converted to a conditional allele after recombination with Flp recombinase (Figure S1A in Supplementary Material). Transcripts are forced to be spliced from exon 1 of the Dnase1l3 gene to the splice acceptor side of a lacZ reporter and terminated by the downstream polyA sides. This should result in a knockout allele, as downstream exons are not transcribed (Figures S1A,B in Supplementary Material). To verify that downstream exons are not transcribed from the knockout-first allele [allele name termed Dnase1l3^tm1a(KOMP)Wtsi^ according to Skarnes et al. (31), abbreviated as Dnase1l3^tm1a^], we used cDNA from MACS-enriched DCs from spleens of homozygous and littermate mice in an RT-PCR reaction using primers located in downstream exons 5 and 7 (Figure S1B in Supplementary Material). As shown in Figure S1C in Supplementary Material, transcripts from the mutated allele were undetectable by this sensitive PCR reaction. This shows that the knockout-first allele is a null allele, as expected from the analysis of many similar alleles generated by the KOMP program (32). We also crossed mice with the knockout-first allele with E2a-Cre deleter mice (33) to obtain an exon 2 knockout allele missing exon 2 which will be termed Dnase1l3^Δex2^.

### Development of Anti-dsDNA Autoantibodies in Dnase1l3-Deficient Mice

**Figure 1:**
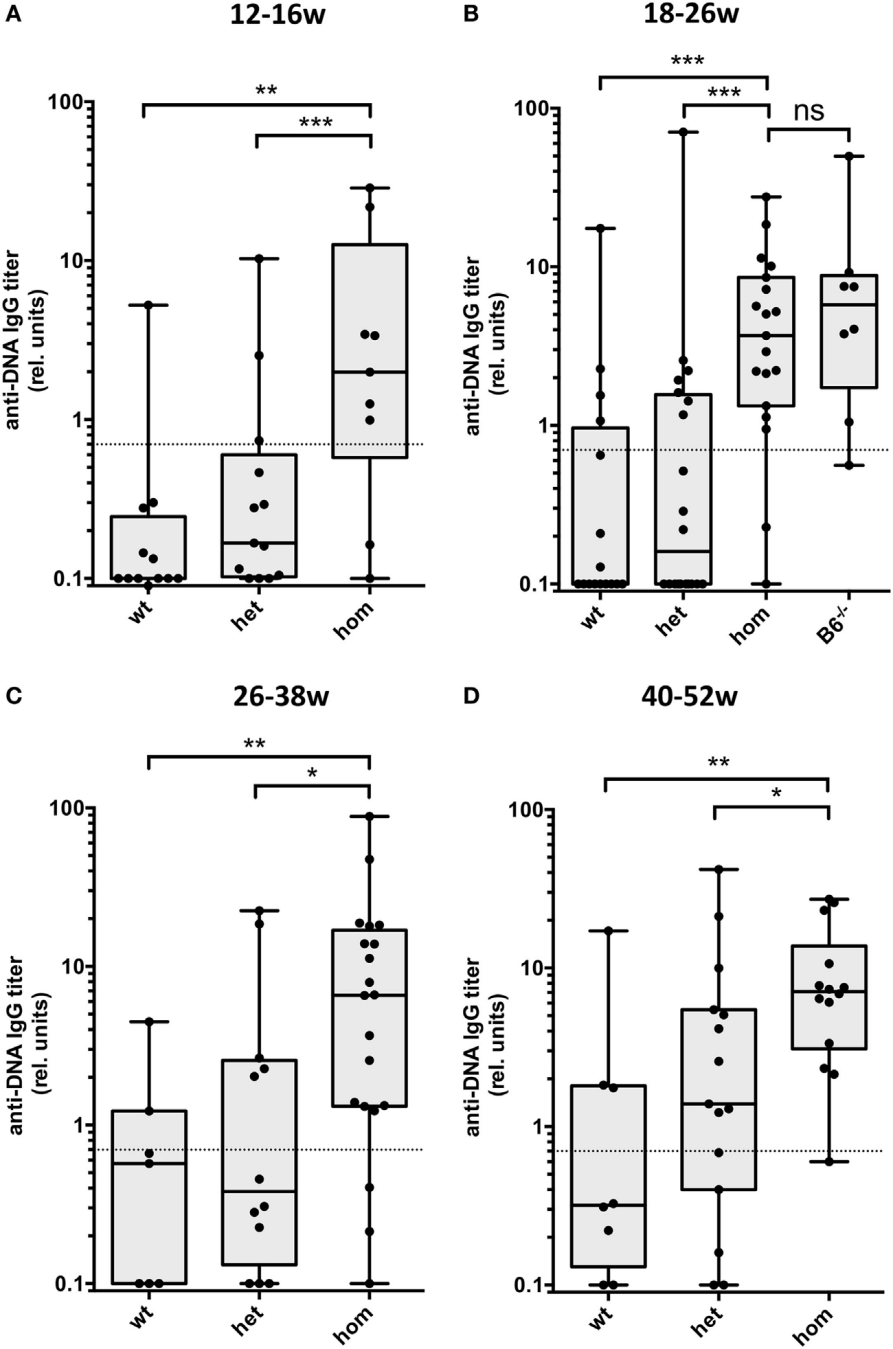
Development of IgG autoantibodies against double-stranded DNA (anti-dsDNA) antibodies in Dnase1l3-deficient mice. Sera from mice at the age of **(A)** 12–16 weeks, **(B)** 18–26 weeks, **(C)** 26–38 weeks, and **(D)** 40–52 weeks were tested for IgG anti-dsDNA antibodies by ELISA. The mice are grouped according to the genotype of the Dnase1l3^tm1a^ mutation. All cohorts of mice are derived from 129 × C57BL/6 mixed background except homozygous mutant mice in **(B)** which are backcrossed to pure C57BL/6 background (B6^−/−^). Relative binding units are presented as compared to a standard serum pool from 9-month-old NZB/W mice. The dotted lines represent a cutoff derived from a panel of sera from C57BL/6 mice. Data are presented as box plot with individual data represented by filled circles (*p < 0.05; **p < 0.01; ***p < 0.001 Mann–Whitney *U* test).

**Figure 2:**
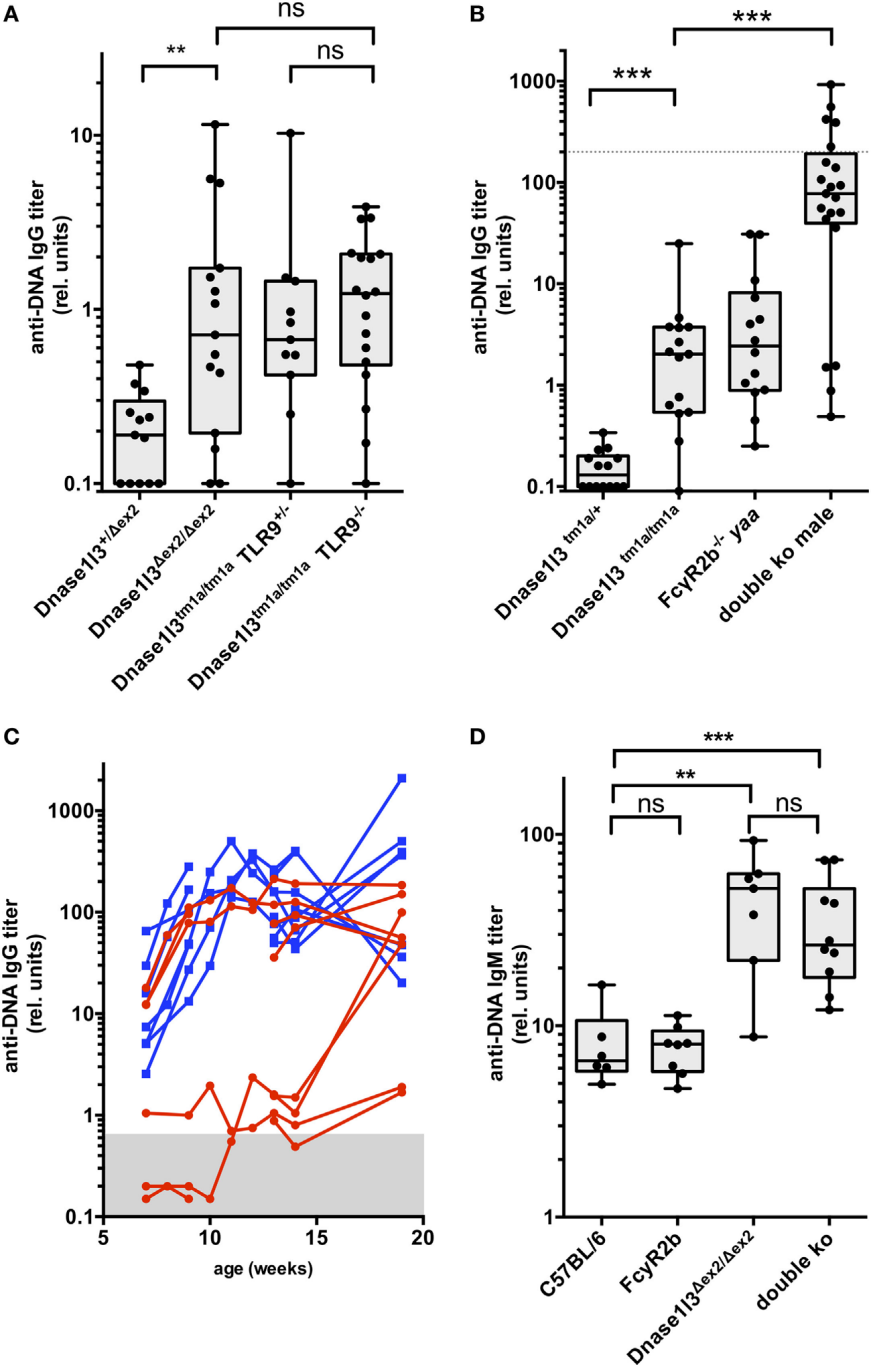
Development of IgG and IgM autoantibodies against double-stranded DNA (anti-dsDNA) antibodies in double-deficient mice. In **(A)**, the results from 3- to 4-month-old toll-like receptor 9 (TLR9) × Dnase1l3 mice are displayed. The genotypes of the mice are denoted at the axis. In **(B)**, the results from 3-month-old FcgR2b × Dnase1l3 mice are displayed. The genotypes of the mice are denoted at the axis. The dotted lines represent anti-dsDNA antibody levels of a serum pool of 9-month-old female NZB/W mice. Data are presented as box plot with individual data represented by filled circles. **(C)** Follow-up of IgG anti-dsDNA autoantibody levels in individual female FcgR2b × Dnase1l3 double-deficient mice (red) and male FcgR2b *yaa* × Dnase1l3 double-deficient mice (blue). In **(D)**, the IgM anti-dsDNA levels from a cohort of 2- to 3-month-old FcgR2b × Dnase1l3 mice are displayed. The genotypes of the mice are denoted at the axis. Data are presented as box plot with individual data represented by filled circles (*p < 0.05; **p < 0.01; ***p < 0.001 Mann–Whitney *U* test).

We analyzed serum IgG anti-dsDNA autoantibodies from groups of mice at different ages. As shown in Figure 1A, homozygous Dnase1l3-deficient mice showed significantly elevated IgG anti-dsDNA antibody levels already at young age (3–4 months). At later time points (Figures 1B–D), these titers show only a moderate increase. Dnase1l3-deficient mice at 1-year of age still have significantly, but modestly increased IgG anti-dsDNA titers. All groups except group 4 in Figure 1B are derived from mice in a mixed 129 × C57BL/6 background. For the group of mice at the age of 18–26 weeks fully congenic C57BL/6 mice have similar anti-dsDNA antibody levels, suggesting that the mixed genetic background of the mice had little if any influence on the development of anti-dsDNA antibodies (Figure 1B). For a cohort of 16- to 28-week-old mice, the influence of the sex on the development of autoantibodies was analyzed. As shown in Figure S2 in Supplementary Material a tendency for higher anti-dsDNA autoantibodies in female versus male mice was observed, which was not statistically significant. In addition, we analyzed homozygous Dnase1l3^Δex2^ mice with a complete deletion of exon 2 of *Dnase1l3* and found similar levels of anti-dsDNA autoantibodies as compared to mice with a homozygous Dnase1l3^tm1a^ mutation, further supporting the notion that Dnase1l3^tm1a^ mutation is a null mutation (Figure 2A). Thus, Dnase1l3-deficient mice spontaneously develop moderate levels of anti-DNA autoantibodies already at 3-month of age which are not increasing significantly upon aging. Neither a significant contribution of residual 129 genetic background nor any significant influence of the sex of the mice influenced anti-dsDNA autoantibodies.

### Epistatic Interactions With Other Mutations Involved in the Generation of Anti-dsDNA Antibodies

As anti-dsDNA autoantibody levels remained moderately elevated in Dnase1l3-deficient mice up to an age of 1 year, we asked the question whether other relevant single mutations might influence anti-dsDNA autoantibody titers. First, we intercrossed Dnase1l3-deficient mice with TLR9-deficient mice to obtain double-deficient mice. As demonstrated in Figure 2A, additional TLR9-deficiency had no significant influence on anti-dsDNA titers in Dnase1l3-deficient mice. We also generated double-deficient mice for *Dnase1l3* and Fcgr2b in which male mice in addition contain the *yaa* mutation accelerating SLE-like disease in the FcγRIIB-deficient background (12). As shown in Figure 2B, we observed extremely high anti-dsDNA titers in double-deficient male mice at the age of 3-month reaching anti-dsDNA levels similar to 9-month-old NZB/W female mice. At this age, FcγRIIB^−/−^ *yaa* male mice had anti-dsDNA titers comparable to single Dnase1l3-deficient mice which were around 50-fold lower than in male double-deficient mice (Figure 2B).

Since these anti-dsDNA antibody levels in double-deficient male mice were higher than in any other SLE-prone strain of mice including MRL/lpr and NZB/W mice at the same age, we weekly analyzed anti-dsDNA antibodies in sera starting at the age of 7 weeks. We included also female mice in this analysis. As shown in Figure 2C, already at the age of 7 weeks all male mice (blue curves in Figure 2C) had clearly elevated anti-dsDNA serum levels that might have be transferred from the double-deficient mothers *via* placental transfer. We consider this unlikely, however, as some female littermate mice at the same age had normal levels of anti-dsDNA antibodies in their sera (Figure 2C). In all male mice of this cohort and in three out of six female mice which we analyzed at this young age, IgG anti-dsDNA antibody levels massively increased within 3–5 weeks up to 50-fold, reaching a maximum level around 10–12 weeks of age. Most of the other female mice developed these maximum levels of autoantibodies at the age of 15–20 weeks. Thus, mutations in *Dnase1l3* and Fcgr2b show strong epistasis and the *yaa* mutation further increases the penetrance of early hyperproduction of anti-dsDNA antibodies in male mice in this model.

As IgM anti-DNA autoantibodies typically develop early in SLE models, we analyzed IgM anti-dsDNA levels in a cohort of 2- to 3-month-old animals of the different genotypes. Interestingly, Dnase1l3 single-deficient mice show highly elevated IgM anti-dsDNA autoantibodies comparable to double-deficient mice, whereas FcγRIIB-knockout mice had normal IgM anti-dsDNA levels (Figure 2D). Thus, loss of tolerance toward DNA is an early event in Dnase1l3-deficient mice.

### Spontaneous Germinal Center Formation and Elevated Production of Anti-dsDNA Secreting Cells in Dnase1l3 and FcγRIIB Double-Deficient Mice

**Figure 3:**
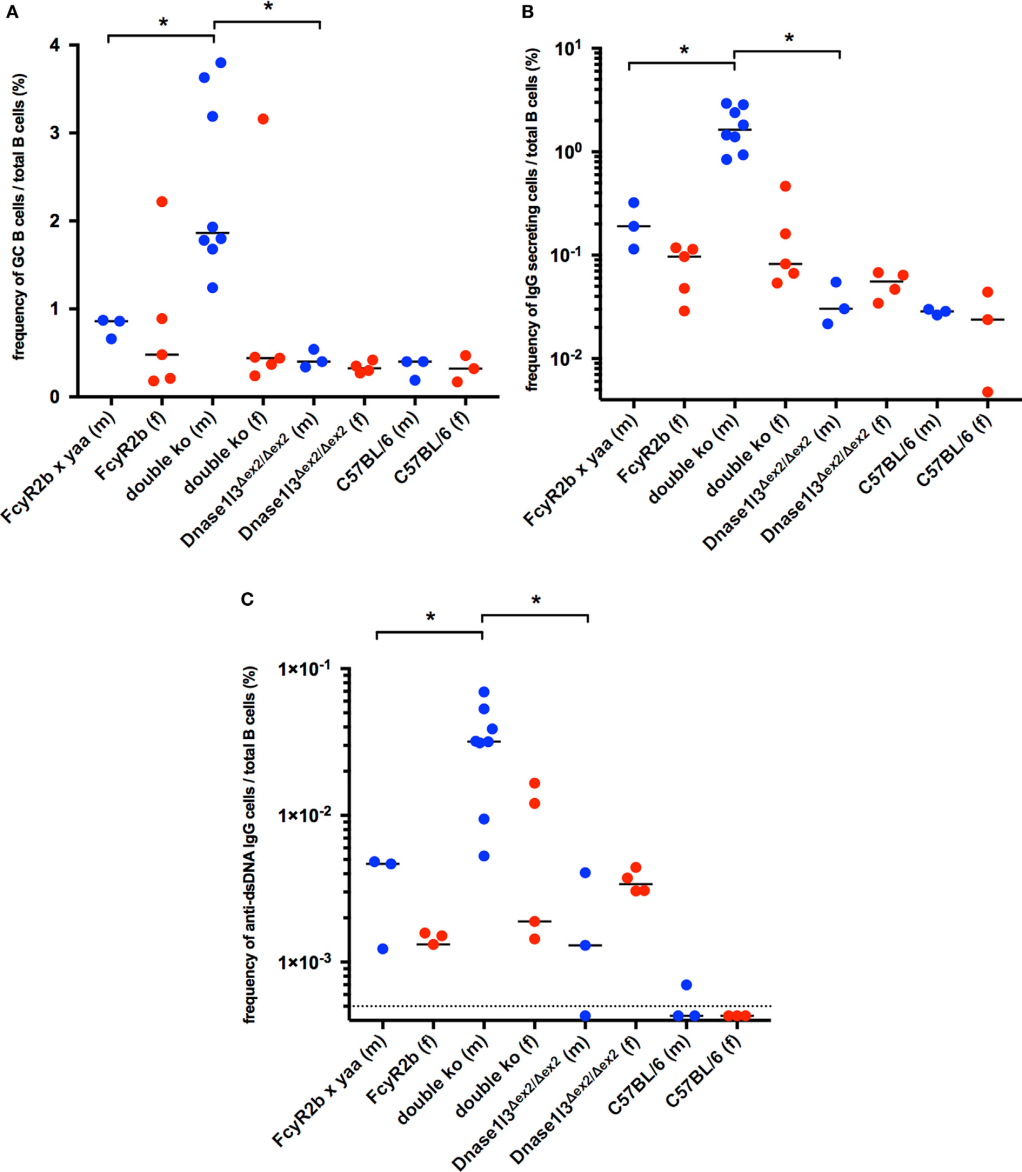
Spontaneous germinal center development and elevated production of autoantibodies against double-stranded DNA (anti-dsDNA) secreting cells in Dnase1l3 and FcgR2b double-deficient mice. **(A)** Analysis of splenic B cells from 9- to 14-week-old mice by flow cytometry. The frequency of GL7^+^, Fas^+^ germinal center B cells among all CD19^+^ B cells is displayed. The frequency of total IgG **(B)** and IgG anti-dsDNA **(C)** secreting B cells was determined by Elispot. Blue circles represent male mice, red circles represent female mice; dotted line in **(C)** represents the limit of detection (*p < 0.05; Mann–Whitney *U* test).

We have proposed earlier that anti-dsDNA antibodies evolve from non-autoreactive progenitors in germinal centers (34, 35) and strong evidence has been accumulated that the FcγRIIB plays an important role in B cell tolerance in the GC (17). We therefore analyzed spontaneous germinal center development in double-deficient mice at the age of 9–14 weeks. As shown in Figure 3A, all male double-deficient mice had elevated frequencies of CD38^lo^, GL7^+^, Fas^+^ GC B cells in the spleen when compared to both single deficient male or female mice or wild-type C57BL/6 mice. In this cohort, one out of five female double-deficient mice showed a dramatically elevated GC B cell frequency. Therefore, the early and strong anti-dsDNA antibody response is accompanied by GC hyperactivation in double-deficient mice.

The activation of B cells in the spleens of double-deficient mice is also accompanied by high frequencies of IgG—antibody secreting plasma blasts or plasma cells (Figure 3B). Up to 3% of all B cells were found to be IgG antibody secreting cells, suggesting a considerable hyperactivation of the B cell compartment. Only a fraction of these IgG secreting B cells was anti-dsDNA specific (0.6–3.8% of all IgG secreting cells, Figures 3B,C).

### Analysis of Somatic Hypermutation in IgG Anti-dsDNA Hybridomas

To gain further insight into the mechanism of anti-dsDNA development in double-deficient mice, we prepared hybridomas from spleens of unimmunized mice and selected IgG anti-dsDNA hybridomas for the sequence analysis of V_H_- and in selected case also V_L_-genes. For this analysis, we focused on female double-deficient mice that were selected for previous rise of IgG anti-dsDNA autoantibodies before fusion. Data for the V_H_-genes are summarized in Table 2. Most hybridoma clones used the IgG2c isotype (25/31 clones) followed by a few IgG2b and one IgG3 anti-dsDNA hybridoma, similar to anti-DNA autoantibodies in other SLE-prone mice (36). In all three mice that were used for the generation of hybridomas we observed clonally related hybridomas clearly derived from one B cell and diversified by somatic mutations. We detected somatic mutations in the heavy chains of all hybridomas (Table 2; Figure S3 in Supplementary Material). In many cases, the numbers of mutations within the V_H_ and V_L_ gene region were relatively low with few replacement mutations present. For those clones that contributed with three or more individual hybridomas, we analyzed the diversification from the germline sequence as well as intraclonal diversification in more detail. Very few shared mutations were observed within the clonal relatives as shown in Figure S3 in Supplementary Material suggesting a “bush-like” diversification by somatic hypermutation from a single B cell rather than a stepwise maturation with intermediate selection.

**Table 2:** Summary of V region gene analysis from autoantibodies against double-stranded DNA hybridomas.

Hybridoma	Clone^a^	Isotype	VH gene	JH gene	Mutations: total/non-silent	CDR3^b^	Number of Arg in CDR3^c^
**Mouse 1, female 14w**							
4B10.1	#1	IgG2c	V5-17	J4	4/3	AR**R**KL**R**NYYAMDY	2
6B9.1	#1	IgG2c	V5-17	J4	3/2	AR**R**KL**R**NYYAMDY	2
7F4.1	#2	IgG2b	V1-82	J2	1/1	ARPG**RR**G**R**YYFDY	3
3C5.2	#2	IgG2c	V1-82	J2	2/0	ARPG**RR**G**R**YYFDY	3
3F5.1	#3	IgG2c	V1-7	J3	4/2	ARSYYGSKGWFTY	0
3E2.1	#4	IgG2c	V1-26	J2	6/2	ASGDSSGPFDY	0
1E7.1	#5	IgG2c	V2-2	J4	1/0	ARN**R**L**RR**GLDY	3
2B11.1	#6	IgG2c	V1-81	J2	9/6	AGEHAGPYYFDY	0
10A12.1	#7	IgG2c	V5-9-1	J3	5/3	TRGGDSSGY**R**FAY	1

**Mouse 2, female 12w**							
1E5	#1	IgG2c	V5-17	J4	1/1	AR**R**GL**R**GVMDY	2
8F4	#1	IgG2c	V5-17	J4	7/5	VR**R**GL**R**GAMDY	2
8H4	#1	IgG2c	V5-17	J4	4/1	VR**R**GL**R**GAMDY	2
1G2.1	#1	IgG2c	V5-17	J4	4/3	AR**R**GL**R**GAMNY	2
2C1.2	#1	IgG2c	V5-17	J4	4/2	VR**R**GL**R**GAMDY	2
4H2.1	#1	IgG2c	V5-17	J4	2/0	AR**R**GL**R**GAMDY	2
5G3	#2	IgG2b	V5-17	J4	6/4	AKQL**R**L**R**YYAMDY	2
6E5	#2	IgG2b	V5-17	J4	4/3	SKQL**R**L**R**YYAMDY	2
9A10	#2	IgG2b	V5-17	J4	6/5	AKQL**R**L**R**YYAMDY	2
3C5	#3	IgG2b	V9-4	J4	1/1	ARDGNSYEGFAY	0

**Mouse 3, female 24w**							
5G12	#1	IgG2c	V1-81	J3	7/6	AEDGYAWFTY	0
5F11	#1	IgG2c	V1-81	J3	4/4	AEDGYVWFAY	0
4C2	#1	IgG2c	V1-81	J3	13/10	AEDGYVWFAY	0
3B7	#2	IgG2c	V1-9	J3	9/4	ARE**R**NYITGFAY	1
1F10	#2	IgG2c	V1-9	J3	9/7	ARE**R**NYITGFAY	1
1D12	#2	IgG2c	V1-9	J3	2/1	ARE**R**NYITGFAY	1
2A2	#3	IgG2c	V7-3	J2	1/1	ARFPAGT**RR**YYFDY	2
5B4	#3	IgG2c	V7-3	J2	4/2	ARFPAGT**RR**YYFDY	2
3A9	#3	IgG2c	V7-3	J2	3/2	ARFPAGT**RR**YYFDY	2
1G1	#4	IgG2c	V5-17	J3	7/6	ARNYYVN**RR**GFAY	2
1G5	#5	IgG2c	V5-17	J3	8/6	TS**R**QL**R**L**RR**VAY	4
3F12	#6	IgG3	V1-26	J3	3/2	TRKGWDDAY	0

*Orange shaded, recurrent VH gene; blau coloured, arginine residues in CDR3*.

*^a^Clonally related hybridomas as defined by identical VH, D, and JH usage, identical CDR3 length and >95% nucleotide identity in CDR3*.

*^b^CDR3 as defined by IMGT (37)*.

*^c^CDR3 as defined by Kabat et al. (38)*.

We found that the CDR3 regions of the heavy chains of the hybridomas have an unusually high frequency of arginines, as it has been noted before in collections of anti-dsDNA hybridomas from SLE-prone mouse strains (39). Whereas 6,203 arginines can be found among 166,000 amino acids (3.7%) in the CDR3 region of mouse antibodies in the large abYsis antibody database,^2^ all anti-dsDNA hybridomas together described here have a frequency of 16.2% (47 arginines among 289 CDR3 amino acids; *p* < 0.001, Chi-Square). Interestingly, we found a recurrent usage of one V_H_ gene (VH5-17) among expanded clones in all three independent mice (shaded in Table 2). In summary, sequence analysis of anti-dsDNA hybridomas showed clear evidence for clonal expansion and somatic mutation among the anti-dsDNA hybridomas from double-deficient mice.

### Early Expansion of T Follicular Helper (T_FH_) Cells in Dnase1l3 and FcγRIIB Double-Deficient Mice

**Figure 4:**
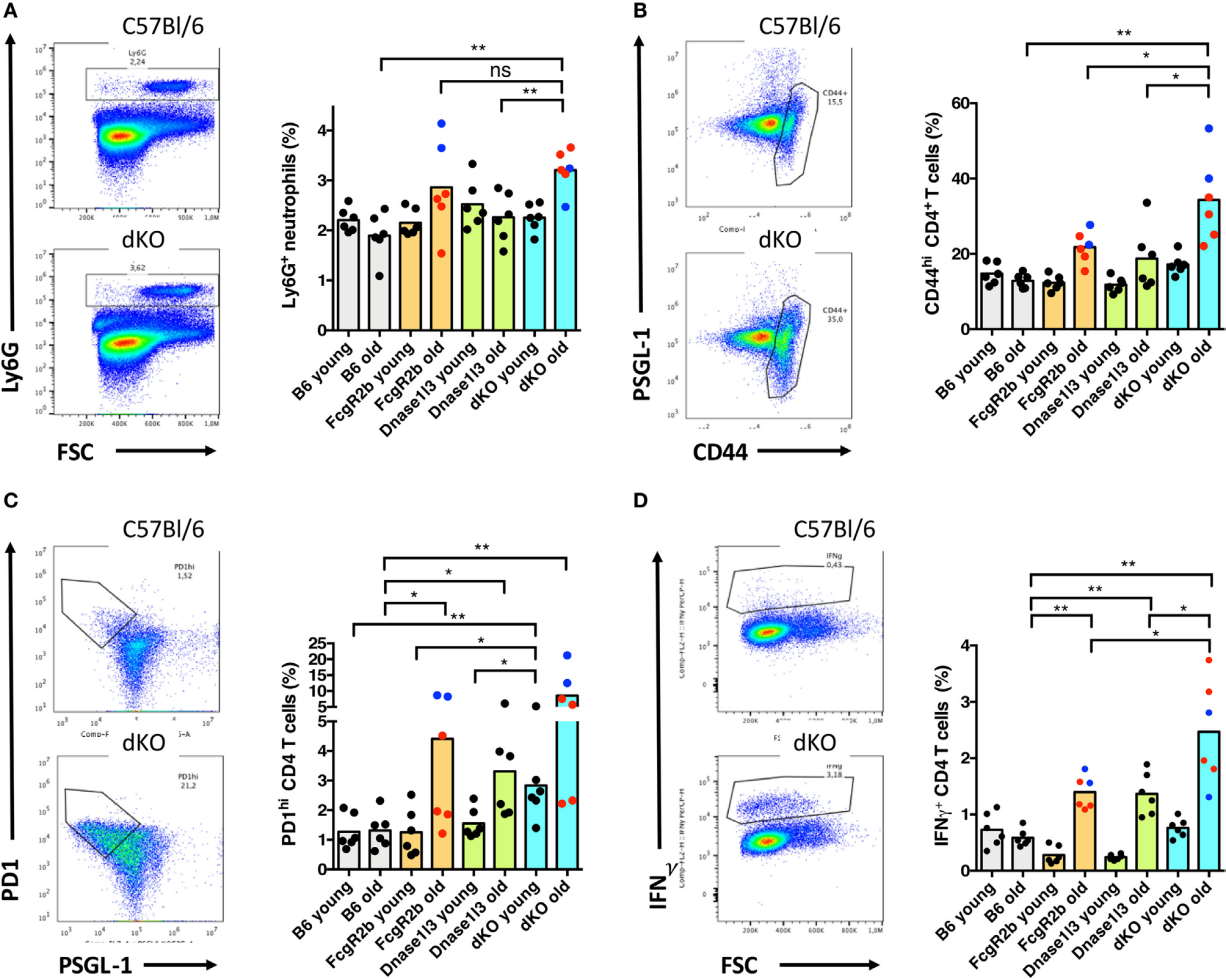
Early expansion of T follicular helper (T_FH_) cells and elevated levels of neutrophils in older Dnase1l3 and FcgR2b double-deficient mice. **(A)** Analysis of the expansion of Ly6G-positive neutrophils in the spleen of young (8–9 weeks old) and older (14–19 weeks old) mice. The genotypes of the mice are denoted at the axis. In **(B)**, activation of the CD4^+^ T cell compartment is displayed by the frequency of CD44^hi^ CD4 cells. **(C)** Analysis of the expansions of PD1^hi^ PSGL-1^lo^ T_FH_ cells. In **(D)**, the frequency of IFN-γ producing CD4^+^ T cells is displayed. As an example for each analysis, a density plot is shown for wild-type and double-deficient mice. Blue circles specifically highlight male mice, red circles represent female mice (*p < 0.05; **p < 0.01; Mann–Whitney *U* test).

To get some mechanistic insight into the early spontaneous formation of germinal centers, we analyzed cohorts of young (8–9 weeks of age) and older mice (14–19 weeks) of single- and double-knockout mice for myeloid cell populations as well as T helper cell subpopulations in the spleen. The analysis of the frequency of monocytes, DCs, plasmacytoid DCs, and macrophages in the spleen did not reveal significant changes among single- or double-mutant mice as compared to C57BL/6 mice of similar age (data not shown). We observed an increase of Ly6G-positive neutrophils in the spleen of older FcγRIIB- as well as double-knockout mice, most likely reflecting inflammatory activity (Figure 4A). General activation of the CD4^+^ T cell compartment as evidenced by high levels of CD44 expression was observed in older FcγRIIB- as well as in double-knockout mice, but interestingly not in older Dnase1l3 single knockout animals (Figure 4B). Expansions of CD44^hi^ T_H_ cells were not observed in any cohort of young mice. T cell hyperactivation was particularly high in male animals carrying the *yaa* allele as denoted by the blue symbols but not restricted to *yaa*-animals in the cohorts (Figure 4B). We observed an expansion of the PD1^hi^ PSGL-1^lo^ T_FH_ cells in older FcγRIIB-knockout mice and particularly striking in double-knockout mice (Figure 4C). Notably, T_FH_ cell are significantly expanded already in young double-knockout mice (Figure 4C). The frequency of IFN-γ producing CD4^+^ T cells was elevated in both single-knockout strains of older age and strongly elevated in older double-knockout animals (Figure 4D). Thus, early expansion of T_FH_ cells in the spleen correlates with the enhanced spontaneous germinal center development and high-level IgG anti-dsDNA production in double-deficient mice.

## Discussion

The results described in this study reveal a strong genetic interaction of two individual susceptibility genes for anti-dsDNA autoantibody production in SLE that have been described in familiar lupus, as well as in knockout mouse models. Null mutations in the *DNASE1L3* gene were described in familial forms of SLE in Saudi Arabia, Turkey, and Italy (19–21). Anti-dsDNA autoantibodies were observed in almost all of the cases reported. In addition, a potentially defunctioning missense SNP present in relatively high frequency in the Caucasian population (40) shows a very high association with anti-centromere antibody-positive systemic sclerosis (41). For FCGR2B a SNP with considerable variation in frequency among human populations results in threonine replacing isoleucine in the transmembrane domain of the receptor (15). This causes the variant receptor to be excluded from sphingolipid rafts, resulting in a malfunctioning receptor (15). As both SNPs have a relatively high frequency within the Caucasian and Asian population (the missense SNP in Dnase1l3 is rare among populations with African origin^3^), we consider it highly worthwhile to analyze the frequency of these two variations among SLE patients. Current GWAS studies can explain only a minority of the heritability of complex diseases, a phenomenon that has been termed missing heritability (42). Epistatic interactions would be difficult to detect in GWAS studies because of computational challenges and low statistical power (43). We are currently analyzing such a possible epistatic interaction of *Dnase1l3* and *Fc*γ*RIIB* in a German/French SLE cohort from the upper Rhine.

In our analysis of cohorts of double-mutant mice, we also introduced the *yaa* allele that results in a *TLR7* gene-duplication (44, 45). The analysis of female double-mutant mice and male double-mutant carrying the *yaa* mutation clearly revealed an influence of *yaa* on the penetrance of development of high levels of anti-dsDNA antibodies very early. Only approximately 50% of female mice developed high levels of anti-dsDNA antibodies at the age of 13 weeks. This is in accordance with the notion that *yaa* accelerates systemic autoimmunity as a genetic modifier in a context of a coexisting SLE background (44, 46). Also, the penetrance of early hyperactivation of germinal centers is much lower in female double-deficient mice, consistent with the described role of the *yaa* allele for defective germinal center selection in SLE (47). Importantly, double-deficient *yaa* male mice at the age of 3 months showed about 40 times higher anti-dsDNA levels when compared to *Fc*γ*RIIb*^−/−^ *yaa* male mice of the same age.

Double-mutant mice showed a hyperactivation of spontaneously developing germinal centers which correlates with an early expansion of T_FH_ cells in the spleen. Expansions of T_FH_ cells were first noted in *sanroque* mutant mice with a lupus-like disease and autoantibody production (48) and later also in some SLE patients with severe disease (49). As T_FH_ cells are essential for germinal center responses (50), our findings here in the context of two epistatically acting mutations in the C57BL/6 genetic background further strengthen the model that dysregulated germinal center reactions are crucially involved in the early phases of the generation of IgG anti-dsDNA autoantibodies and the development of SLE (35, 50).

In *Fc*γ*RIIB*-deficient mice, an increase in anti-DNA reactive GC B cells was observed and somatic mutations contributed to the generation of highly autoreactive IgG antibodies (13). Opposite to the findings in single *Fcgr2b-*deficient mice, however, we observed clonal expansions of anti-DNA reactive B cells as well as concomitant expansion of anti-DNA plasmablasts or plasma cells. These combined observations prompt a further development of our model of the evolution of anti-DNA autoantibodies in germinal centers (35). FcγRIIB and potentially other negative regulators for B cell signaling (51) might play a major role in the regulation of autoreactive GC B cells which otherwise would develop from non-self-reactive or low-level self-reactive precursors by somatic mutations (34). We now propose that the deficiency to eliminate nuclear material would drive such autoreactive B cell from the germinal center into clonal expansion and plasma cell differentiation, presumably outside of the germinal center. Our sequence analysis points in the direction that these B cells mutated only for limited periods of time. The “bush-like” diversification by somatic hypermutation from a single B cell rather than a stepwise maturation with intermediate selection is compatible with this model. Similar “bush-like” genealogies were also observed in SLE-prone mice (52, 53). An extraordinary high content of arginine residues in the CDR3 region of the heavy chain, which is also observed in our study here, apparently is a very common prerequisite for such an evolution of anti-dsDNA antibodies (39). Recent work attributed removal of apoptotic microparticles from the circulation as a major role of Dnase1l3 to prevent loss of tolerance to chromatin (22). We suggest an alternative explanation which is mutually not exclusive and might operate at local sites, particularly in the liver, the spleen, and lymph nodes. We found the highest levels of Dnase1l3 expression in cells with excellent antigen-presenting competence, high expression of scavenging receptors and TLRs, i.e., DCs and Kupffer cells in the liver. Local secretion of Dnase1l3 might function as a kind of shield to prevent these cells from being activated by damage-associated molecular patterns *via* TLRs and other receptors (54, 55).

In the same direction, local Dnase1l3 secretion might eliminate or lower the activation of DCs after uptake of DNA-containing immune complexes *via* Fc receptors which has the potential to trigger maturation and interferon production of DCs (17). Under non-inflammatory conditions, the inhibitory FcγRIIB would dominate activating signals from activating Fcγ receptors on DCs (17), however. This could reflect the situation in our single Dnase1l3-deficient mouse, in which anti-dsDNA production is stalled at relatively moderate levels. When FcγRIIB is blocked on human DCs, high levels of IL-12p70 can be induced when antibody coated tumor cells are delivered to the DCs (56) and type I interferon is induced after delivery of immune complexes to DCs (57). This scenario might explain the phenotype of Dnase1l3/FcγRIIB double-deficient animals described here. Under these circumstances, any DNA-containing immune complex could potentially trigger DC maturation and production of type I interferon and stimulate T_H_1 driven responses. A self-perpetuating feedback loop would result when anti-dsDNA autoantibodies have evolved in such a scenario (58). For the initial trigger, IgG anti-dsDNA antibodies are not an essential content in the stimulating immune complexes. Pathogen-derived DNA together with pathogen-specific antibodies would be sufficient for starting such a DC-initiated self-perpetuating feedback loop.

Our conditional Dnase1l3 allele as well as conditional deletion of FcγRIIB will allow the detailed analysis of the cell types that are essential for the protection against uncontrolled evolution of anti-dsDNA autoantibodies in germinal centers. Potentially, comprehensive understanding of this regulation might allow the development of new targeted therapies for SLE.

## Ethics Statement

Animal care and experiments were approved by the animal ethics committee of Regierung von Mittelfranken (Animal Ethics Number: 54-2532.1-21/09 and TS-05/5).

## Author Contributions

TW and BN performed experiments, analyzed data, and interpreted data. AS, NE, DS, and AA performed experiments and analyzed data. THW performed experiments, conceived the experiments, analyzed data, interpreted data, and wrote the manuscript. All authors were involved in critically reading of the manuscript and approved the final version to be published.

## Conflict of Interest Statement

No potential conflict of interest relevant to this publication is reported. The reviewer, RM and the handling Editor declared their shared affiliation.

## Footnotes

^1^www.imgt.org (Accessed: April, 2016–March, 2018).

^2^http://www.bioinf.org.uk/abysis3.1 (Accessed: November 12, 2017).

^3^http://phase3browser.1000genomes.org (Accessed: February 15, 2018).
