# Supplementary material for: Epistatic Interactions Between Mutations of Deoxyribonuclease 1-Like 3 and the Inhibitory Fc Gamma Receptor IIB Result in Very Early and Massive Autoantibodies Against Double-Stranded DNA
Source: Front Immunol. 2018 Jul 5;9:1551. doi: 10.3389/fimmu.2018.01551 (PMC6041390; doi:10.3389/fimmu.2018.01551)
Supplement: Supplementary file 1 [file data_sheet_1.docx]

Supplementary Material

Epistatic interactions between mutations of Dnase1like3 and the inhibitory Fc gamma receptor IIB result in very early and massive anti-dsDNA autoantibody production

**Thomas Weisenburger, Bettina von Neubeck, Andrea Schneider, Nadja Ebert, Daniel Schreyer, Andreas Acs, Thomas H. Winkler***

*** Correspondence:** Thomas H. Winkler: Thomas.winkler@fau.de

# Supplementary Data

Oligonucleotide primers used in this publication summarized in an Microsoft Excel sheet (primers.xlsx)

# Supplementary Figures and Tables

**Supplementary Figure 1.** **Targeting strategy and analysis of Dnase1l3 deficient mice**

(A) Schematic overview of targeting strategy used by the KOMP “Knockout First” strategy. The abbreviations of the names of the alleles described in this study are shown in the boxes (modified from Skarnes et al., 2011). (B) In scale graphic representation of the wildtype (upper) and the Dnase1l3 knockout-first allele (lower) generated by the targeting strategy. Splicing is implied by the dotted lines. Oligonucleotide primers (black arrows) used for the amplification of cDNA from the wt and knockout-first allele spanning intron 5 and 6 are depicted. (C) Agarose gel representing PCR amplification of exons 5 to exon 7 of *Dnase1l3* from cDNA isolated from enriched dendritic cells from one litter of mice with the genotypes denoted above the lanes of the gel. Amplification products of HPRT cDNA are shown in the upper part of the panel. In mice with a homozygous knockout-first mutation, cDNA for the 3´ coding part of Dnase1l3 are undetectable, characterizing the mutation as a null allele.

**Supplementary Figure 2.** **Anti-dsDNA autoantibodies in female versus male Dnase1l3 deficient mice.** IgG anti-dsDNA autoantibody levels were determined in 16-24 week old female or male Dnase1l3-deficient mice. Data from 16 male and 16 female mice are shown, data from homozygous Dnase1l3^tm1a^ and Dnase1l3^∆ex2^ mice were pooled. The levels of antibodies are not significantly different (Mann Whitney, p=0.09)


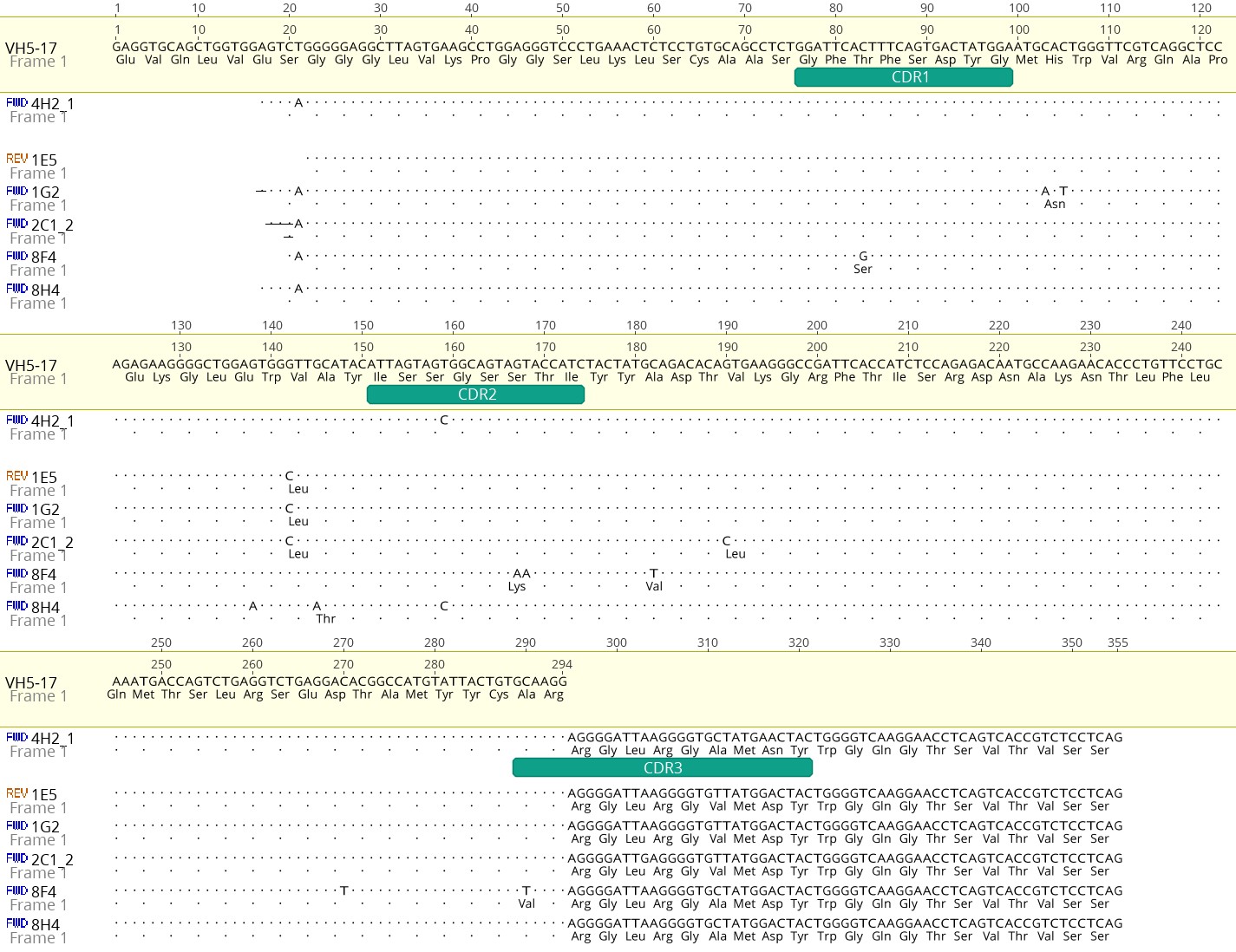


mouse 2, clone #1 HC

mouse 2, clone #1 LC


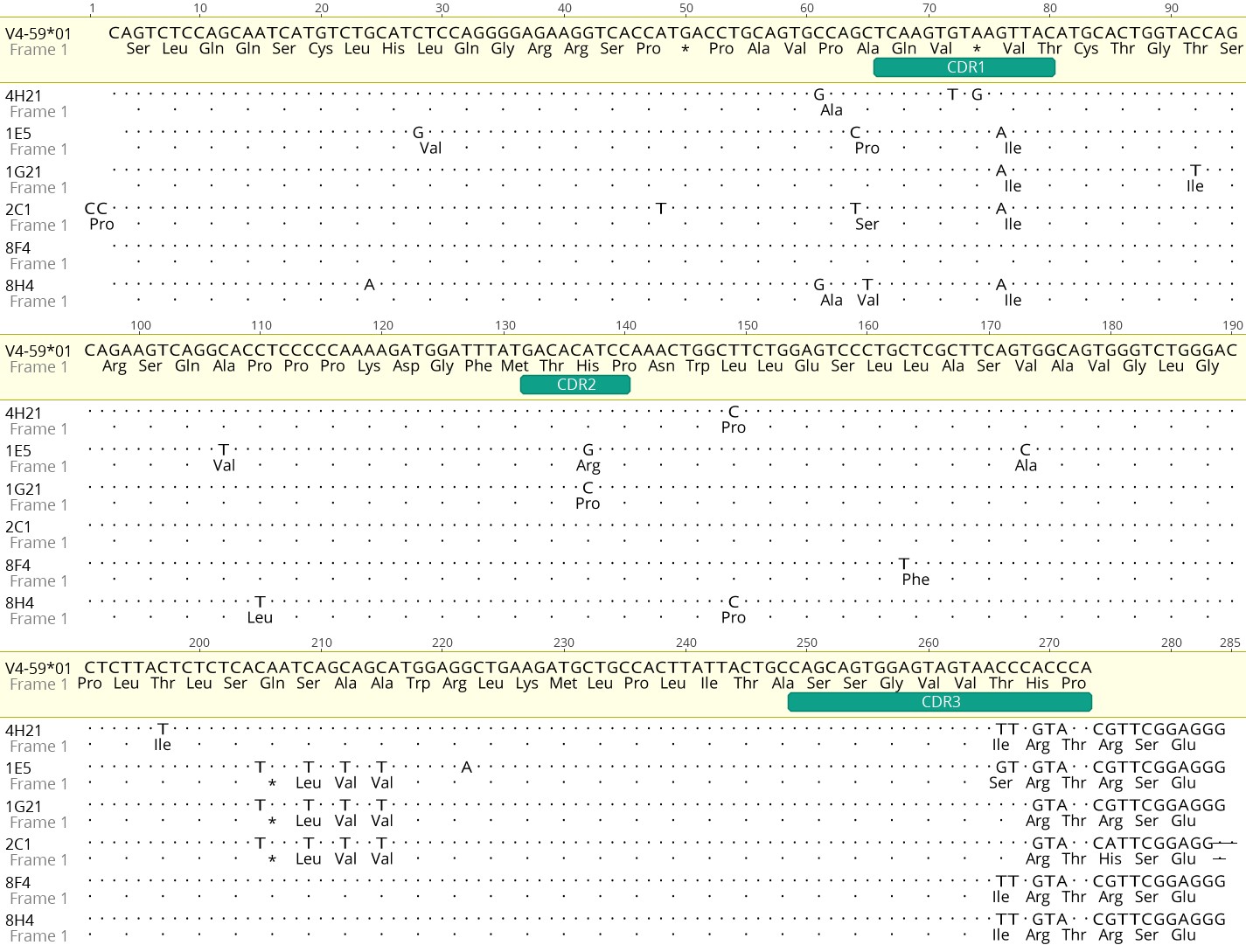


mouse 2, clone #2 HC


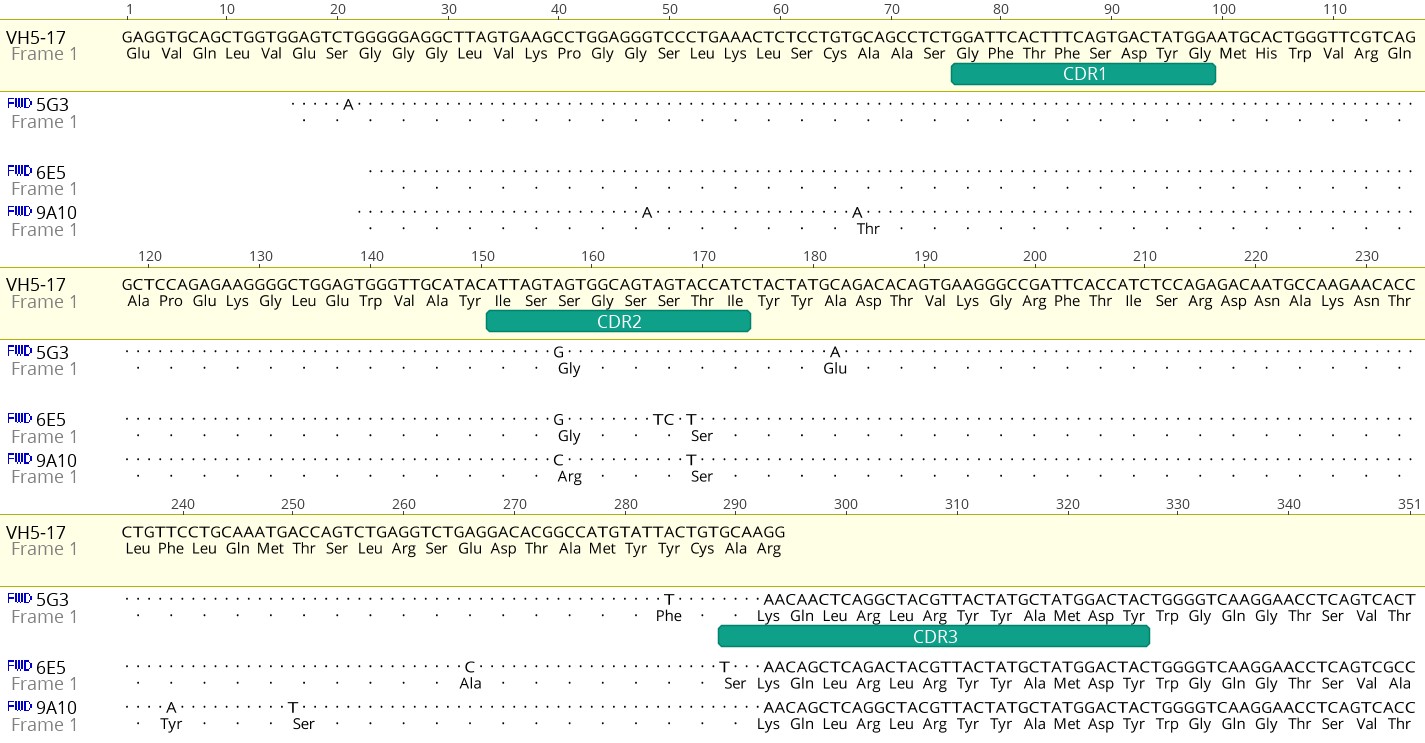


mouse 3, clone #1 HC


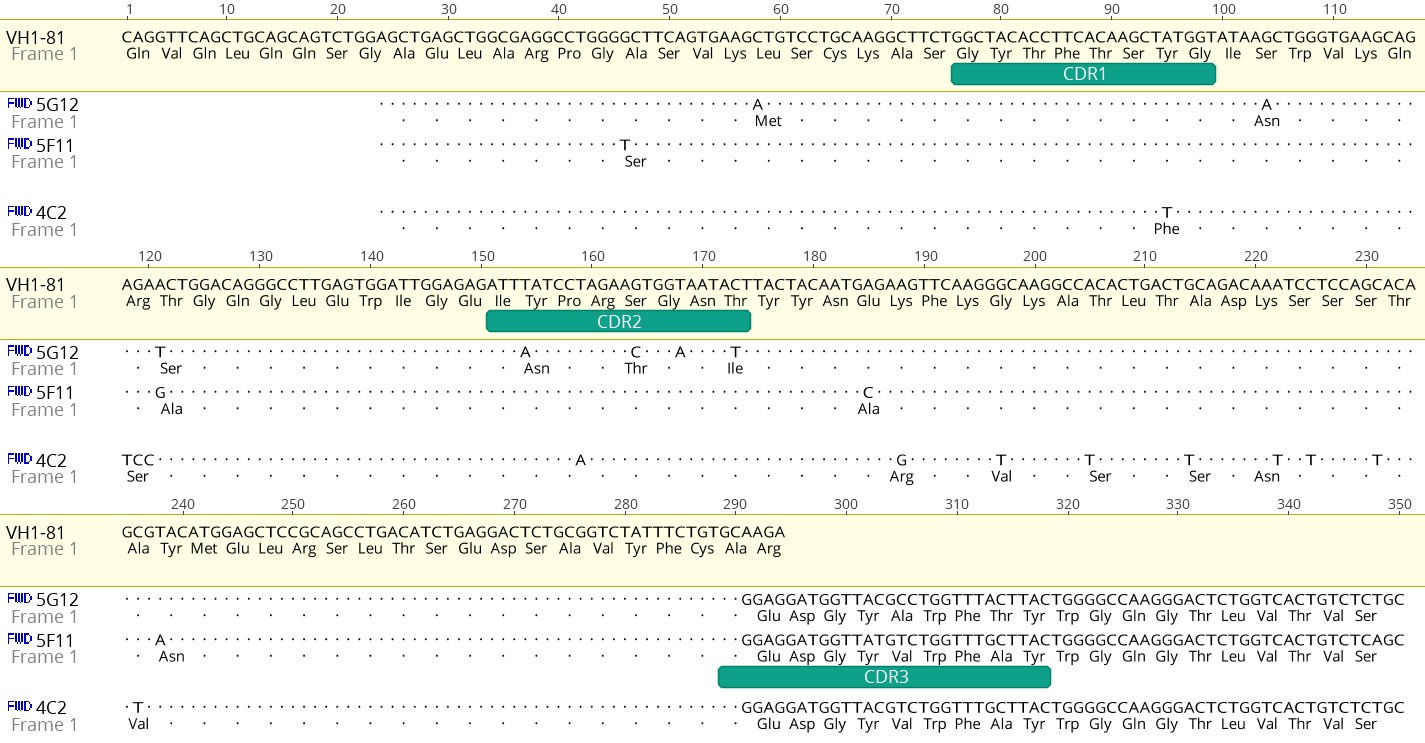


mouse 3, clone #2 HC


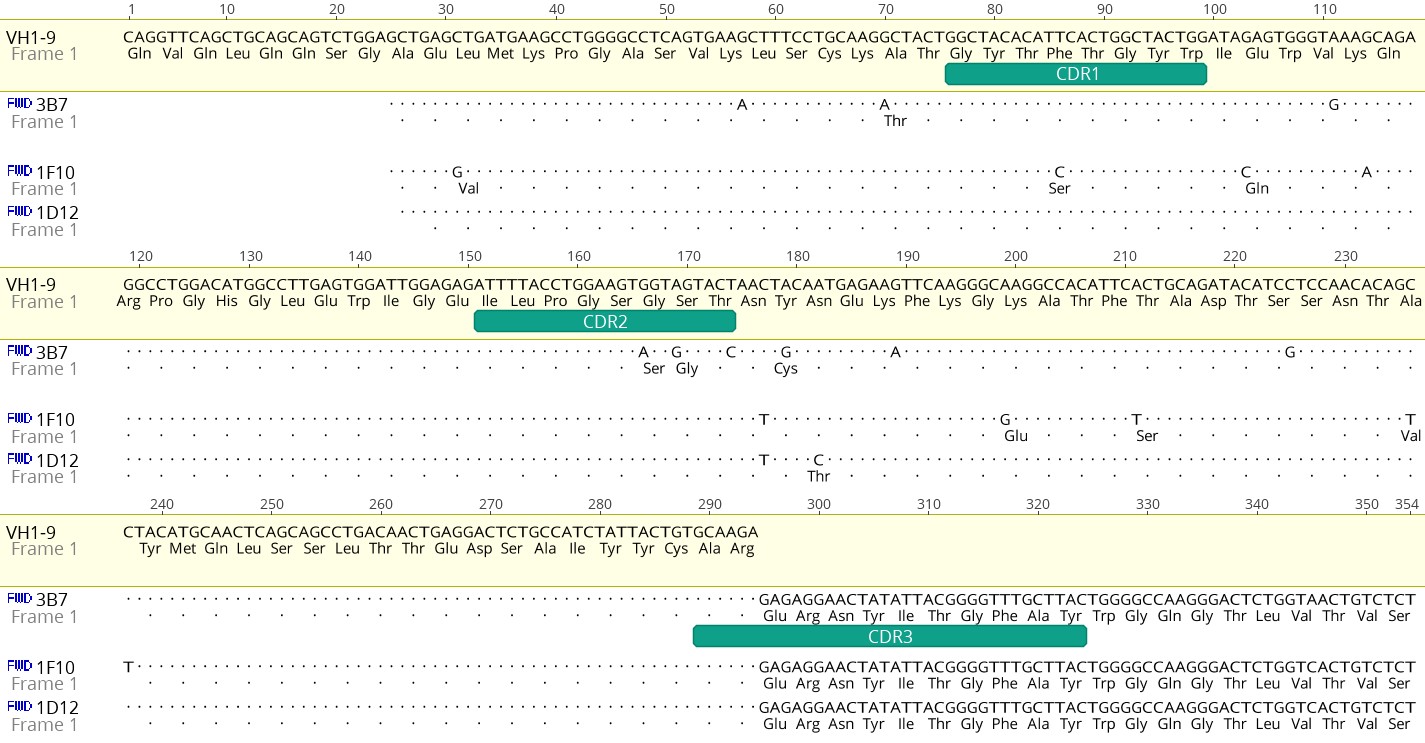


mouse 3, clone #3 HC


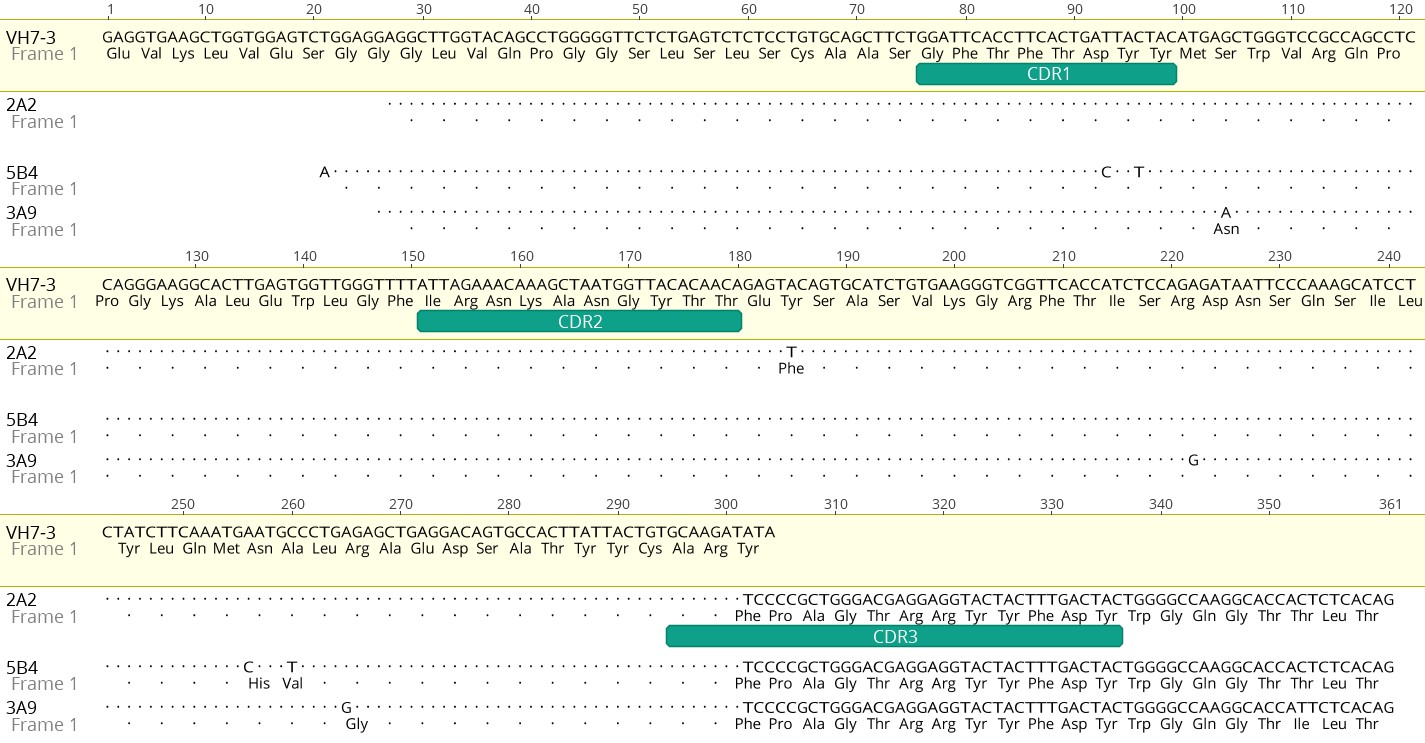


**Supplementary Figure 3. Alignment of V_H_ and V_L_ gene segments from clonally expanded B cell clones.** Clones with 3 or more members as displayed in Table 2 (mouse number, clone number) are aligned to the germline V_H_ and V_L_ genes as analyzed by the IMGT V-QUEST tool. Replaced nucleotides as well as amino acids are displayed. HC: heavy chain; LC: light chain
